# Sparse prediction informed by genetic annotations using the logit normal prior for Bayesian regression tree ensembles

**DOI:** 10.1002/gepi.22505

**Published:** 2022-11-09

**Authors:** Charles Spanbauer, Wei Pan, ADNI, The Alzheimer's Disease Neuroimaging Initiative

**Affiliations:** ^1^ Division of Biostatistics University of Minnesota Minneapolis Minnesota USA

**Keywords:** ensemble learning, genetics, high‐dimensional prediction, sparsity

## Abstract

Using high‐dimensional genetic variants such as single nucleotide polymorphisms (SNP) to predict complex diseases and traits has important applications in basic research and other clinical settings. For example, predicting gene expression is a necessary first step to identify (putative) causal genes in transcriptome‐wide association studies. Due to weak signals, high‐dimensionality, and linkage disequilibrium (correlation) among SNPs, building such a prediction model is challenging. However, functional annotations at the SNP level (e.g., as epigenomic data across multiple cell‐ or tissue‐types) are available and could be used to inform predictor importance and aid in outcome prediction. Existing approaches to incorporate annotations have been based mainly on (generalized) linear models. Bayesian additive regression trees (BART), in contrast, is a reliable method to obtain high‐quality nonlinear out of sample predictions without overfitting. Unfortunately, the default prior from BART may be too inflexible to handle sparse situations where the number of predictors approaches or surpasses the number of observations. Motivated by our real data application, this article proposes an alternative prior based on the logit normal distribution because it provides a framework that is adaptive to sparsity and can model informative functional annotations. It also provides a framework to incorporate prior information about the between SNP correlations. Computational details for carrying out inference are presented along with the results from a simulation study and a genome‐wide prediction analysis of the Alzheimer's Disease Neuroimaging Initiative data.

**Abbreviations**
ADNI: Alzheimer's Disease Neuroimaging Initiative
BART: Bayesian additive regression trees
MCMC: Markov chain Monte Carlo
PG: pólya‐gamma
SNP: single‐nucleotide polymorphism

## 1 INTRODUCTION

Prediction with high‐dimensional data is both important and challenging. For example, in statistical genetics it is of great interest to predict a complex trait or disease using high‐dimensional genetic information, such as single‐nucleotide polymorphism (SNP) data. Such a prediction model, called polygenic risk scores (PRS), for a complex disease can be a useful prognostic for disease prevention and treatment development (Pattee & Pan, 2020). Alternatively, predicting gene expression is a necessary first step to illuminate (putative) causal genes in transcriptome‐wide association studies (TWAS) (Gamazon et al., 2015; Gusev et al., 2016), which motivated and will be the focus of this study as shown in our real data example. Due to the high‐dimensionality (e.g., the number of predictors/SNPs can be large relative to the sample size), weak signals (i.e., the effect sizes of the SNPs are quite small), and high correlations among local/nearby SNPs, the problem is quite challenging. However, there may be additional information about the predictors that can be useful in extracting a usable signal. For SNPs in particular, recent large‐scale functional epigenomics studies, such as the NIH ENCODE and Roadmap Epigenomics projects, provide rich resources to characterize functional consequences of SNPs, especially those in noncoding regions.

Using such functional annotation data could inform and prioritize predictor importance and is an exciting prospect in statistical genetics that is an active area of research (Liu et al., 2020). There may be a relationship between the annotation profile of an SNP and its importance as a predictor of the outcome. These functional annotations represent multiomic data obtained from various cell‐ and tissue‐types and can be high‐dimensional. Most importantly, for a specific problem, some of these annotations are expected to be (weakly) informative while others are not, but which annotations are informative and which are not is in general unknown.

A popular way to annotate an SNP is to specify whether it is an expression quantitative trait locus (eQTL) (Chen et al., 2016; Lu et al., 2016), which may or may not be useful depending on whether the corresponding gene and the trait being considered are related. Thus, treating all eQTL SNPs equally may not be the most efficient for a given trait. Furthermore, the existing approaches are mainly based on generalized linear models. How to effectively incorporate high‐dimensional functional annotations into more flexible nonparametric/nonlinear modeling is an exciting frontier with potential to improve the overall performance in many genetic contexts where prediction is desired, motivating our real data analysis.

Regression tree ensembles have been an increasingly popular method for obtaining reliable and high‐quality predictions for nonlinear relationships. In particular, Bayesian Additive Regression Trees (BART) has shown a great deal of potential because of its sample prediction accuracy and avoidance of overfitting (Chipman et al., 1998, 2010). BART, as its name suggests, is based on a framework of Bayesian probability and so inference is carried out using Markov‐Chain Monte Carlo (MCMC) sampling (Tierney, 1994). This probability framework means that the uncertainty estimates and intervals for any unknown quantity, including transformations of unknown quantities, are readily available from the posterior distribution. This is in contrast to other regression tree methods. Additionally, extending a BART model to handle more complicated data or incorporate additional flexibility is usually possible, sometimes trivially so, with a hierarchical Bayesian specification, as done in this article.

BART, in the traditional formulation, however, may have difficulty if the data exhibit sparsity. The prior probability used for predictor choice within the splitting rule is not flexible enough for sparse situations and can display poor behavior. This is shown in Linero (2018) who also offers a method for increasing the flexibility of this probability. The method modifies the standard BART prior, which sets equal the probability of predictor selection in the splitting rule, to instead be modeled using a Dirichlet distribution (DART). This yields a regression tree ensemble that is adaptive to predictor sparsity and allows it to focus on the important predictors while ignoring the unimportant ones. However, the Dirichlet prior is somewhat inflexible, requiring that the variable selection probabilities are “almost” independent.

**Figure 1:**
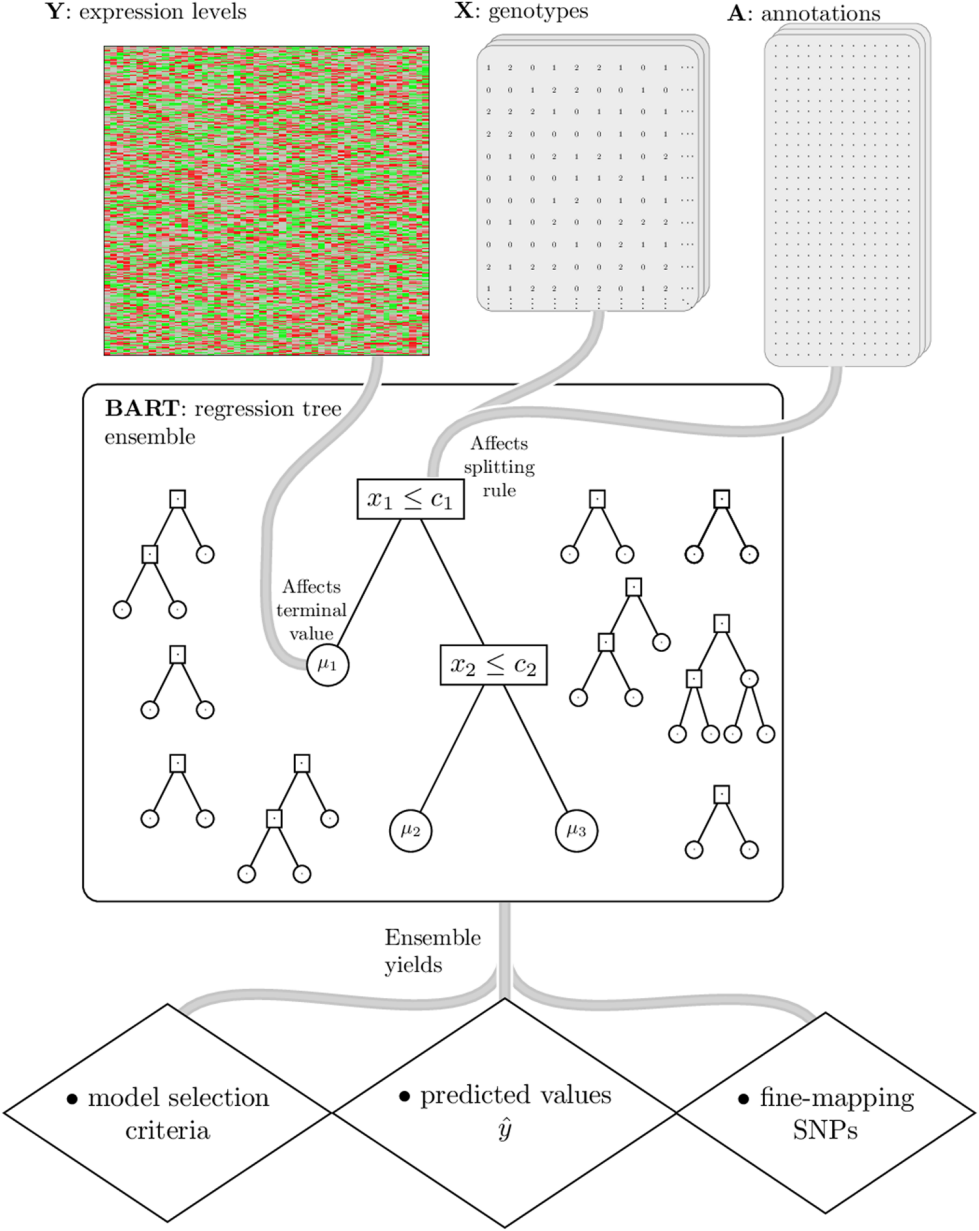
Depiction of the workflow for performing a genome‐wide scan using BART while also incorporating the functional annotations. Note how the outcome Y affects the terminal node values, while the predictors and annotations, X and A, affect the splitting rules for the interior nodes.

Aiming to improve the performance with our real data, this article presents the logit normal prior as an alternative to the standard BART prior or Dirichlet prior. The logit normal allows for correlations between the inclusion probability of the predictors to be incorporated, assuming prior information. Additionally, because of its relationship to the normal distribution, the logit normal prior is a natural choice to incorporate the functional annotations using a hierarchical logit link. Therefore, this prior can solve both problems at once: the inflexibility of the Dirichlet distribution and accounting for the functional annotations. Finally, this prior is computationally tractable because the posterior distribution can be sampled efficiently using the Pólya‐gamma (PG) augmentation technique of Polson et al. (2013), similar to the augmentation strategies for probit (Albert & Chib, 1993) and logistic (Held & Holmes, 2006) links. The overall idea of the methodology as applied to this article is presented in Figure 1. The outcomes y inform the terminal node values while the predictors X and the functional annotations A inform the splitting rules in the interior nodes. Taken together this model can provide the posterior distribution for outcome predictions $\hat{y}$, Bayesian model selection criteria, and the fine‐mapping of SNPs for prediction using the characteristics of the tree ensemble.

The logit normal prior is incorporated into the overall BART algorithm, resulting in a nonlinear prediction method that is grounded entirely in Bayesian probability. Furthermore, this prior can be incorporated into BART for any type of outcome including probit, logistic, survival (Sparapani et al., 2016), competing risks (Sparapani et al., 2020), recurrent events (Sparapani et al., 2020), and repeated measures/random effects (Spanbauer & Sparapani, 2021; Tan et al., 2018) resulting in broad applicability of this method.

In Section 2, a brief overview of the BART and DART methods is presented. In Section 3, the logit normal prior and its MCMC sampling strategies are developed. Section 4 presents a simulation for the methodology and Section 5 presents its application to genome‐wide prediction and demonstrates its usefulness. Finally, these results and future work are discussed in Section 6.

## 2 BAYESIAN ADDITIVE REGRESSION TREES OVERVIEW

BART is a Bayesian nonparametric ensemble learning method for nonlinear regression. In the continuous case, BART seeks to model regression relationships of the form

$$y_{i}=f\left(x_{i}\right)+\epsilon_{i},$$

for i=1,…,n, where $x_{i}=\left(x_{i1},\text{…},x_{ip}\right)\prime$ and $\epsilon_{i}\overset{\mathrm{iid}}{∼}N\left(0,\sigma^{2}\right)$. The quantity of interest to be estimated in this case is simply $f\left(x_{i}\right)=E\left[y_{i}|x_{i}\right]$. In the case of binary outcomes $y_{i}\in \left\{0,1\right\}$, a probit or logistic link can be used so that $f\left(x_{i}\right)$ can be transformed to the scale of $E\left[y_{i}|x_{i}\right]=P\left(y_{i}=1|x_{i}\right)\in \left(0,1\right)$.

In the following section, the prior specification and posterior sampling for BART are briefly discussed with emphasis placed on the predictor choice prior. The interested reader can refer to Chipman et al. (2010) for a more detailed treatment of BART.

### 2.1 BART priors and MCMC inference

The two unknown quantities in the BART model are f and $\sigma^{2}$. Because these quantities are unknown, MCMC sampling will be performed and inference carried out on the sampled posterior distribution.

The variance prior uses a scaled inverse chi‐squared distribution, alternatively parameterized as the inverse‐gamma distribution. The degrees of freedom hyperparameter is set to a reasonable value (i.e., integers from 1 to 5), while the scale parameter is set so that a rough data‐based estimate of $\sigma^{2}$ is at the 0.95 percentile of the prior distribution, to scale the before plausible values of $\sigma^{2}$ within the context of the data, though other percentiles can be specified as necessary. Alternatively, cross‐validation can be performed to select both the degrees of freedom and percentile. The data‐based estimate can be an estimate of $\sigma^{2}$ in a linear regression model or the sample variance of the outcome itself. In general, the sample variance is used when the number of coefficients to be estimated eclipses the number of observations and so it is not possible to fit the linear model.

**Figure 2:**
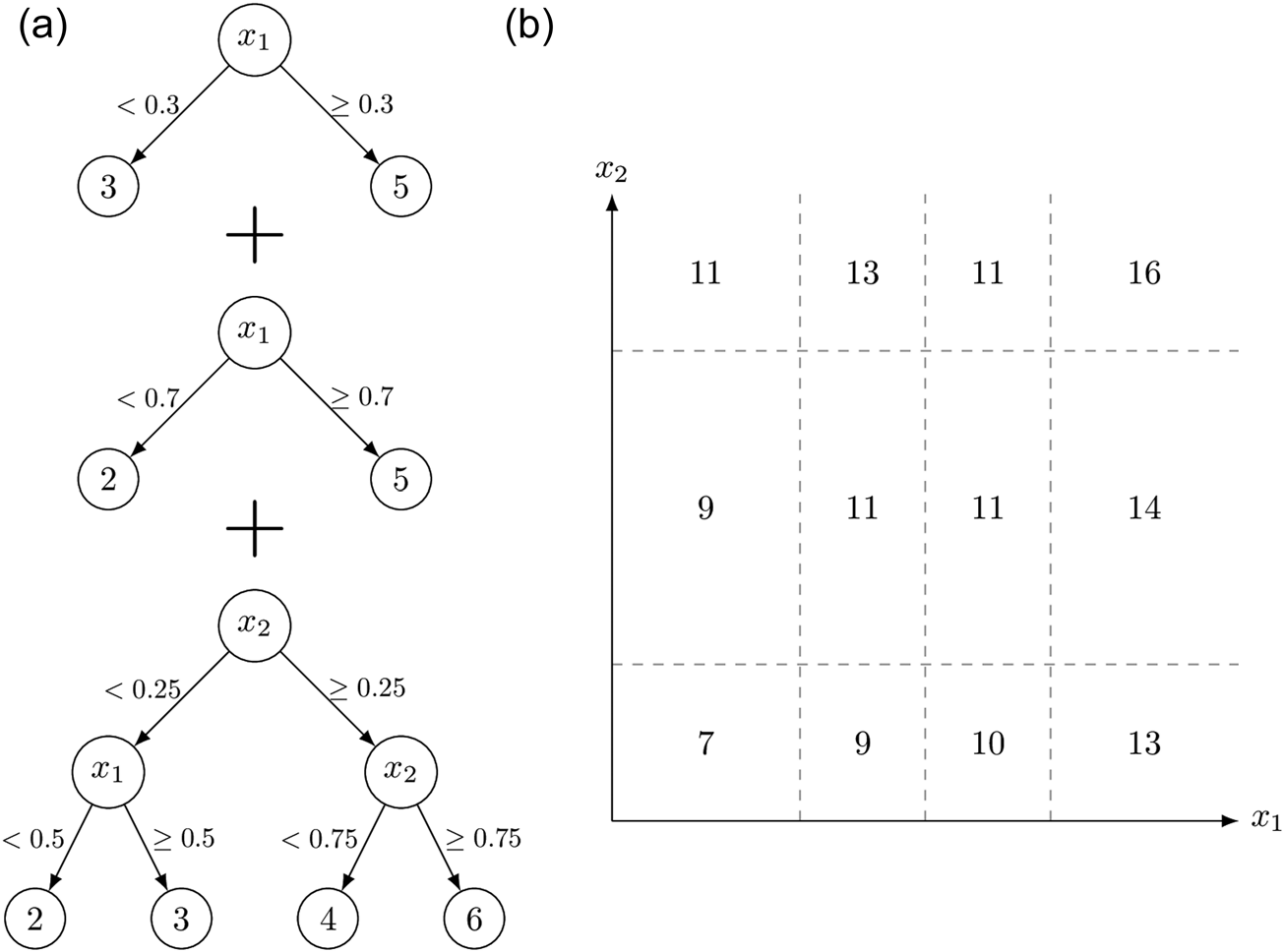
Toy example depicting a regression tree ensemble with H=3 along with the resulting partition of the predictor space. The numbers on the right are the values in each partition of the predictor space after adding the appropriate terminal node values on the left. The horizontal lines at 0.25 and 0.75 represent the partitions in $x_{2}$ while the vertical lines at 0.3, 0.5, and 0.7 represent the partitions in $x_{1}$. This demonstrates how regression tree ensembles can reliably estimate nonlinearity and interaction.

The prior on f is represented as the sum of H constant piecewise‐defined functions called regression trees. Regression trees recursively partition the predictor space into regions inside each of which the expected value of the outcome is estimated. This assumption is formulated as

$$f\left(x_{i}\right)\approx \sum_{h=1}^{H}g(x_{i};T_{h},{ℳ}_{h}),$$

where h=1,…,H is indexing the trees in the ensemble, $T_{h}$ represents the partition of the x space as defined by tree h, and ${ℳ}_{h}$ represents the terminal nodes at the bottom of tree h. A toy example of this is given in Figure 2. This prior is further simplified by assuming that each tree is independent of the other trees. Placing a prior on f now reduces to simply placing a prior on each $\left(T_{h},{ℳ}_{h}\right)$ pair for all h. This pair is decomposed into $T_{h}$ marginally and then ${ℳ}_{h}|T_{h}$ because the number of terminal nodes depends on the tree structure. This same prior is applied to all H trees.

There are three major components to the prior on $T_{h}$, that is, the structure of the tree. These include the probability of a node splitting at a particular depth, the probability of which predictor to select for use in the splitting rule, and the probability of selecting a particular cutpoint of the selected predictor for the splitting rules. In many cases, the default settings described below work very well. However, in certain scenarios, such as predictor sparsity with high‐dimensional data, modifying one or more default settings can be useful.

The probability of a node splitting at a particular depth defaults to $\delta \left(d\right)=\alpha {\left(1+\lambda \right)}^{-d}$, where d represents the depth of a particular node starting at 0 for the head node. By default, α=0.95 and λ=2. Note that the default values of δ(d) are set in such a way that the trees are kept shallow, for example, trees at or beyond depth 3 are rare, though splits at the top of the tree are common. Keeping the individual trees from explaining too large a portion of the outcome variability is how the BART prior performs regularization. Regularizing guards against overfitting because, while the entire ensemble can explain a large portion of the variability in the outcome, the individual trees cannot. As such, predictions from the entire ensemble have a lower variance than single tree models, thinking in terms of the bias‐variance tradeoff.

The probability of selecting a particular cutpoint is set to be equal within an equidistant grid for each predictor. There are other contexts where choosing this in a different way can be helpful, but in this context the default behavior is acceptable. Note that, from a computational standpoint, binary predictors can be treated exactly the same as continuous ones. However, nominal predictors with more than one category will need to be transformed into binary dummy predictors. Ordinal predictors can be treated as continuous or nominal depending on the situation.

The final component of the regression tree is the choice of predictor for each interior node. Because this is the focus of the modifications described in the article, we introduce some notation. Let $v=\left(v_{1},\text{…},v_{R}\right)\prime$ represent the indices of the chosen covariates at each interior node that is indexed by r=1,…,R. The vector $s=\left(s_{1},\text{…},s_{p}\right)\prime$, where $\sum_{j=1}^{p}s_{j}=1$ defines the probability of predictor choice. For standard BART, $s_{j}=1∕p$ for all j. In this way, each predictor is considered equally important. However, note that it places zero prior probability at any other point, that is, $P\left(s_{j}=1∕p\right)=1$, so the resulting value of $s_{j}$ in the posterior distribution cannot be anything other than 1∕p for all j. The ensemble is then unable to adapt itself to any predictor importance that could be learned from the data because of this restrictive prior. Section 2.2 describes the Dirichlet distribution as an alternative (Linero, 2018).

The terminal node values in tree h are represented by the vector ${ℳ}_{h}=\left(\mu_{h,1},\text{…},\mu_{h,L_{h}}\right)\prime$, where $L_{h}$ is the number of terminal nodes in the tree. Given a tree structure $T_{h}$, the ${ℳ}_{h}$ elements utilize a normal prior centered around 0 with a standard deviation that scales with the number of trees to take into account the fact that the terminal node values of the ensemble are being summed. This is again reflective of the BART prior regulating each tree so that it only explains a small portion of the variability in the outcome.

The entire MCMC algorithm for drawing f can then be described in two steps, repeated across the trees. For h=1,…,H: draw $T_{h}$ using Metropolis–Hastings conditional on the other trees and then draw ${ℳ}_{h}|T_{h}$ using conjugate Gibbs conditional on the other trees. The residual $y_{i}-\sum_{h'\neq h}g\left(x_{i};T_{h'},{ℳ}_{h'}\right)$ is used to condition on all trees other than tree h (as well as the data y), reflecting the additive nature of the tree ensemble. This is known as “Bayesian backfitting.”

### 2.2 Dirichlet prior for variable selection using BART

There are a few different approaches to handling sparsity using BART, most of which involve the makeup of the splitting rules in the regression tree ensemble. One strategy to solve this problem is to reduce the number of trees H, thereby forcing the regression tree ensemble to only utilize those predictors that have the strongest association with the outcome. However, reducing the number of trees can lead to a reduction in predictive performance. There are also methods based on deriving a permutation distribution for the makeup of the regression tree ensemble after reducing the number of trees (Bleich et al., 2014), but the permutations increase the computational burden. In general, these methods rely on non‐Bayesian quantitative measures that are computed post‐hoc. Ultimately both of these methods are unsatisfying solutions if one requires both prediction and variable selection in an analysis. BART with a Dirichlet prior (DART) is a computationally tractable solution that can do prediction and variable selection simultaneously while not straying from the framework of Bayesian probability and MCMC inference (Linero, 2018). Spike‐and‐slab type priors are another Bayesian solution that have been used within the BART framework (Ročková & van der Pas, 2017).

DART is useful in the case of predictor sparsity because the regression tree ensemble is able to adapt to the important predictors while ignoring the unimportant ones. The Dirichlet prior is also convenient because it is conjugal to the multinomial distribution so that adapting the MCMC algorithm to use this prior is trivial. This can be seen by considering the counts of splits on each predictor throughout the ensemble. Let r=1,…,R index the interior nodes of the ensemble and let $v_{r}\in \left\{1,\text{…},p\right\}∼\text{Mult}\left(1,s\right)$, also defined in Section 2.1, be the choice of predictor for node r. Define $c=\left(c_{1},\text{…},c_{p}\right)\prime$ as the vector of index counts with elements $c_{j}=\sum_{r=1}^{R}I\left(v_{r}=j\right)$, where I(⋅) represents the indicator function. Then c∣s~Mult(R,s) and s∼Dir(θ∕p,…,θ∕p) implies that $s|c∼\text{Dir}\left(\theta ∕p+c_{1},\text{…},\theta ∕p+c_{p}\right)$. Here, θ is a global sparsity parameter. In the DART method, the prior of the sparsity parameter is set so that θ∕(θ+ρ)∼β(a,b) where ρ=p usually. This is equivalent to placing a β‐prime prior scaled by ρ on θ itself (or equivalently, a standardized β‐prime prior on θ∕p if ρ=p).

## 3 THE LOGIT NORMAL PRIOR FOR SPARSITY

The Dirichlet prior does not allow the components of s to have a flexible correlation structure. Because of this, each predictor is chosen at the expense of the others which may be unhelpful, particularly if collinearity is an issue. The logit normal distribution is an attractive alternative because it has the flexibility to model this type of correlation structure and it provides a natural framework for incorporating the functional annotations. Additionally, this prior can easily be used in conjunction with the existing BART MCMC sampling strategy. While the MCMC scheme is slightly more involved than that of the Dirichlet prior, it is still a computationally tractable procedure using augmented PG sampling (Polson et al., 2013). In this section, an overview of the logit normal distribution, the hierarchical formulation of the model that incorporates the annotation data, and directions on how to incorporate this prior into BART are all given.

### 3.1 Logit normal overview

**Figure 3:**
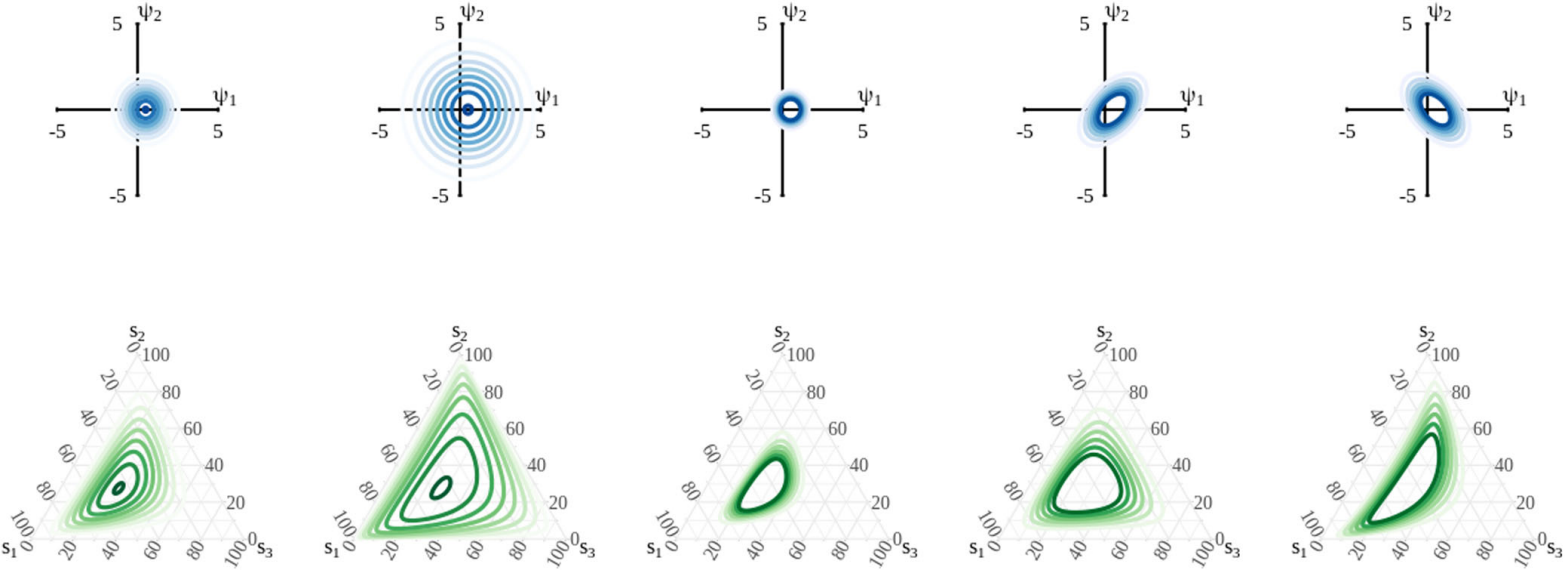
Contours for the pdf of a normally distributed bivariate random variable ψ in the top row and the corresponding contours from the pdf of a three‐category logit normal random variable s. For all five columns the mean of the normally distributed variables is (0.5,0). The variances for the first three columns are 1, 4, and 0.25, respectively and the two elements of ψ are independent. For the fourth and fifth column, the correlation between the two elements of ψ is 0.5 and −0.5, respectively, with variance of 1 for both.

For the logit normal prior, the splitting probabilities are now defined as

(1) $$s_{j}=\frac{\text{exp}\left(\psi_{j}\right)}{1+\sum_{j^{\prime }\neq p}\text{exp}\left(\psi_{j^{\prime }}\right)},$$

where the p‐length vector $\psi =\left(\psi_{1},\text{…},\psi_{p}\right)\prime$ follows a multivariate normal distribution. The name “logit normal” becomes clear from Equation (1): the logit of s gives you a normal variate, in this case, ψ. Note that to avoid identifiability problems, $\psi_{p}=0$, though the choice of p as this index is arbitrary. The way the density of the logit normal is affected by the density of the underlying normal random variate is shown in Figure 3.

However, the logistic likelihood induced by Equation (1) combined with a normal prior leads to a posterior distribution without closed form (Bishop, 2006). Accordingly, data augmentation using the PG distribution can be used to perform MCMC inference through Gibbs sampling (Polson et al., 2013). The most common application of this is Bayesian logistic regression. However, this sampling strategy is possible whenever a model specifies a logit link hierarchically, including a dichotomous logit link as defined above. See Section 3.3 for details.

### 3.2 Incorporating functional annotations

The annotations are incorporated into the model through the expectation of $\psi_{j}$. In the case without annotations, let $\psi_{j}=\eta_{j}$, where $\eta_{j}∼N\left(0,\tau^{2}\right)$ with $\eta_{p}=0$ for identifiability. Here, $\eta_{j}$ represents the random noise of predictor j in terms of its variable selection probability. In this case, $\psi_{j}∼N\left(m_{j0},\tau^{2}\right)$ with $m_{j0}=0$.

To incorporate a vector of annotations for predictor j, call it $a_{j}=\left(a_{j1},\text{…},a_{jT}\right)\prime$, let $\psi_{j}=\beta_{0}+a_{j}^{\prime }\beta +\eta_{j}$ for $\beta =\left(\beta_{1},\text{…},\beta_{T}\right)\prime$, where T is the total number of annotations. The interpretation of $\beta_{t}$ is the increase in ψ per one unit change in annotation t. Again, for identifiability, note that $\psi_{p}=0$ and $\eta_{p}=0$. The ramifications of this are discussed in Section 3.3.3. Of course, any predictor index j∈{1,…,p} can be chosen without loss of generality. Now let $\psi_{j}∼N\left(m_{j0},\tau^{2}\right)$, but $m_{j0}=\beta_{0}+a_{j}^{\prime }\beta$ to take the annotations into account.

As with DART, the method learns about s through c and v which themselves are learned from the original data and the relationship between the outcome and predictors. Under the logit normal prior, the method learns about ψ (and hence s) through c and v in a similar manner. However, by learning about ψ and conditioning on the annotations A, we can learn about β (and the variance term $\tau^{2}$). This is how the annotation data is incorporated into the model.

Note that in this formulation of the prior, the elements of ψ are specified as independent. However, it is also possible to specify a correlation structure for ψ in the prior. This can be done through a stick‐breaking formulation of the dichotomous likelihood (Linderman et al., 2015). For statistical genetics, this may be useful to model the linkage disequilibrium between SNPs if such prior information is available. In this way, $\eta \sim N{\;}_{p-1}\left(0_{p-1},\tau^{2}\Sigma_{\psi 0}\right)$ where the covariance matrix $\Sigma_{\psi 0}$ is specified by the analyst. This could be useful when sets of predictors are known to be correlated with regard to their inclusion probability. It is unlikely that the single realization of vector ψ would be informative enough to estimate their general covariance structure. However, incorporating such structure a priori is possible using the above framework. As an example, one could use an autoregressive structure based on genetic location for $\Sigma_{\psi 0}$.

### 3.3 MCMC sampling

The unnormalized posterior distribution for this model is a multinomial logistic likelihood multiplied by a multivariate normal distribution on ψ, which is obviously nonconjugate. This system defines a true probability distribution, but is not tractable without expanding the likelihood using auxiliary variables. These auxiliary variables allow the unknown parameters ψ to be estimated using a Gaussian update so that the exact posterior can be sampled. Many methods for sampling such a posterior involve auxiliary variables, but this method is almost always more efficient (Polson et al., 2013) than other auxiliary MCMC methods such as Frühwirth‐Schnatter et al. (2009), Gramacy and Polson (2012), and Held and Holmes (2006).

#### 3.3.1 The PG distribution

The basic identity that allows for this auxiliary sampling scheme to work is

(2a) $$\frac{{\left(e^{M}\right)}^{a}}{{\left(1+e^{M}\right)}^{b}}=2^{-b}e^{\kappa M}\int_{0}^{\infty }e^{-\omega M^{2}∕2}p\left(\omega \right)d\omega$$

(2b) $$=2^{-b}e^{\kappa M}E_{\omega }\left[e^{-\omega M^{2}∕2}\right]$$

where ω∼PG(b,0) and κ=a−b∕2. Equations (2a) and (2b) imply that logistic likelihoods can be represented as mixtures of normal distributions with respect to the PG distribution. In particular, note that Equation (2b) represents an "exponential‐tilting” of ω which results in a PG(b,M) random variable. Therefore, Bayesian linear models with a hierarchical logit link function can be sampled using conjugate Gibbs updating, provided there is an easy way to sample from the auxiliary PG(b,M) random variates. Fortunately, such a sampler using an acceptance–rejection algorithm exists due to the BayesLogit R package (Polson et al., 2019).

#### 3.3.2 PG augmentation for sampling $\psi_{j}$

In terms of the logit normal prior described in this article, the likelihood is dichotomous where each predictor represents a “category.” The data are represented by the counts of the splitting rules in the regression tree ensemble $c_{j}$ with $R=\sum_{j=1}^{p}c_{j}$. According to Held and Holmes (2006), the dichotomous likelihood as applied to this model is

(3) $$L\left(\psi_{j}|\psi_{-j},c\right)=\frac{{\left(e^{\psi_{j}-\phi_{j}}\right)}^{c_{j}}}{{\left(1+e^{\psi_{j}-\phi_{j}}\right)}^{R}}$$

with $\phi_{j}=\log\left(\sum_{j^{\prime }\neq j}e^{\psi_{j^{\prime }}}\right)$. Note that $\phi_{j}$ depends only on $\psi_{-j}$ and not $\psi_{j}$. Applying Equations (2a) and (2b), sampling the auxiliary PG variate can be done as $\omega_{j}|\psi_{j},\psi_{-j},c∼\text{PG}\left(R,\psi_{j}-\phi_{j}\right)$.

To sample from $\psi_{j}|\omega_{j},\psi_{-j},c$, Equation (2b) is multiplied by the prior distribution of $\psi_{j}$. Because this step conditions on $\omega_{j}$, the expectation evaluates to its argument. Also, note that $\kappa_{j}=c_{j}-R∕2$. Therefore, the posterior distribution of $\psi_{j}$ can be written as a product of exponentials, and completing the square will yield a Gaussian update for $\psi_{j}$ given by

(4) $$\psi_{j}|\psi_{-j},\omega_{j},c∼N\left(m_{j},V_{j}\right),$$

where $V_{j}=\frac{\tau^{2}}{\omega_{j}\tau^{2}+1}$ and $m_{j}=\frac{m_{j0}+\tau^{2}\left(\kappa_{j}+\omega_{j}\phi_{j}\right)}{\omega_{j}\tau^{2}+1}$.

#### 3.3.3 Gibbs update for the annotation model

The information in the annotations is incorporated using a hierarchical regression model with a logit link. First, note that the model here is $\psi_{j}=\beta_{0}+a_{j}^{\prime }\beta +\eta_{j}$ and both $\psi_{p}=0$ and $\eta_{p}=0$ for identifiability. This implies that the regression hyperplane at point $a_{p}$ passes through $\psi_{p}=0$. Such a model can be estimated by centering the annotations around $a_{p}$. Compute ${\tilde{a}}_{j}^{}=a_{j}-a_{p}$ so that ${\tilde{a}}_{p}=0_{T}$ and set $\beta_{0}=0$. Then, perform no‐intercept regression on the centered annotations to estimate β which is unchanged in interpretation after this transformation. Then the model becomes $\psi_{j}={\tilde{a}}_{j}\beta +\eta_{j}$, though it can be shifted back to the original scale of the annotations to compute $\beta_{0}$ if desired.

Let $\tilde{A}$ denote the (p−1)×T matrix of annotations. Let $\psi_{-p}$ denote the vector of $\psi_{j}$ where j≠p and let ${\tilde{A}}_{-p}$ denote the matrix $\tilde{A}$ where row p is omitted. To estimate β we use a normally distributed and independent prior as $\beta \sim N{\;}_{T}\left(0_{T},v_{\beta 0}I_{T}\right)$ where $v_{\beta 0}$ is some constant in the diagonal variance–covariance matrix. Of course, the prior for β can also be correlated, but that is not considered here. Then, sampling β can be done as

(5) $$\beta |\psi_{-p},\tau^{2},{\tilde{A}}_{-p}∼N{\;}_{T}\left(m_{\beta },V_{\beta }\right)\text{,}$$

where $V_{\beta }={\left(\frac{v_{\beta 0}{\tilde{A}}_{-p}^{\prime }{\tilde{A}}_{-p}+\tau^{2}I_{T}}{v_{\beta 0}\tau^{2}}\right)}^{-1}$ and $m_{\beta }=V_{\beta \left(\tau^{-2}{\tilde{A}}_{-p}^{\prime }\psi_{-p}\right)}$.

Estimating $\tau^{2}$ is also simple with two reasonable choices of prior. Either the traditional inverse‐gamma prior on $\tau^{2}$ or the scaled half‐T prior on τ can be used. The scaled half‐T distribution is equal to the scaled absolute value of a T random variable. These priors are well‐discussed in the Bayesian community (Gelman, 2006; Polson & Scott, 2012). The scaled half‐T distribution has heavier tails, allowing for a higher degree of sparsity in the prior. Additionally, the limit of the density function (from the right) goes to a finite nonzero value for τ=0 with the scaled half‐T, allowing it to plausibly revert back to nonsparse situations. The authors recommend the scaled half‐T prior because of these considerations. The posterior MCMC update will rely on $RSS=\sum_{j=1}^{p-1}{\left(\psi_{j}-{\tilde{a}}_{j}^{\prime }\beta \right)}^{2}$ and is trivial to derive in either case.

The software is given by an R package, entitled sparseBART at https://github.com/cspanbauer/sparseBART. This implementation is based on two previously existing packages: bayesLogit and BART3 which efficiently implement the PG (Polson et al., 2019) and BART (Sparapani et al., 2021) samplers respectively. As such, there is essentially no significant computational differences between traditional BART, the Dirichlet prior of DART, and the logit normal prior of this article. The exception to this is when the number of annotations T becomes large. This is because sampling ψ requires inversion of a T×T matrix at each MCMC iteration.

## 4 SIMULATION

A simulation study was used to evaluate the effectiveness of the different priors among the BART prediction methods, as well as other prediction methods. The methods used are the logit normal prior w/annotations (LN‐A), the logit normal prior w/o annotations (LN‐0), DART, BART, random forest (RF) (Breiman, 2001), and support vector machine (SVM) (Cortes & Vapnik, 1995). The criteria used to evaluate out‐of‐sample prediction accuracy is $R^{2}$. The simulation was modeled after gene SLC24A4.

The true model is $y_{i}=f\left(x_{i}\right)+\epsilon_{i}$, where $\epsilon_{i}\overset{\mathrm{iid}}{∼}N(0,V\left(f\left(x_{i}\right)\right)$, where $V\left(f\left(x_{i}\right)\right)$ is determined via Monte‐Carlo simulation of $x_{i}$ and its transformation using the true f. This implies that the true function f explains half of the variability in the outcome. For the predictors, $x_{i}=\left(x_{i1},\text{…},x_{ip}\right)\prime$, where $x_{ij}\in \left\{0,1,2\right\}$ for i=1,…,n and j=1,…,p, mimicking SNP data for the gene SLC24A4. The SNP minor allele frequencies as observed in the ADNI data from Section 5 are used to simulate these predictors and make up a mix of rare and common variants. The number of predictors is varied as a simulation setting so that p∈{100,250,529}, where 529 is the total number of SNPs in the *cis*‐region for SLC24A4 in the ADNI data set after adjusting for quality. Accordingly, the sample probabilities from real genetic data are used to inform the generation of the simulated data. The correlation structure of the SNPs is also varied between strong and weak correlations. For strongly correlated SNPs, the correlation is simulated as a draw from the inverse‐Wishart $\left(p,I_{p}\right)$ distribution where $I_{p}$ represents the p×p identity matrix. For weakly correlated SNPs, the correlation is simulated as a draw from the inverse‐Wishart $\left(10p,I_{p}\right)$ distribution. T=30 annotations are used for the p simulated *cis*‐SNPs in gene SLC24A4. The annotations used in this simulation and those used in the ADNI real data analysis of Section 5 are a subset of the full set given by Pickrell (2014).

Then, the $\psi_{j}$ are simulated from the model specification according to $\tau^{2}\in \left\{1,5,10\right\}$ which yields three simulation settings: antisparse, moderately sparse, and extremely sparse. While there is considerable variability between simulation iterations, the antisparse setting results in at least 50% of the p SNPs to have some predictive effect, the moderately sparse setting results in roughly 5% to have some predictive effect, and the extremely sparse setting results in less than 1% of them to have some predictive effect on the simulated gene expression.

The true values of β form two simulation settings. The first is a setting where the annotations do not influence the variable importance, that is, $\beta_{t}=0$ for t=1,…,T. The second is a setting where the annotations are informative where $\beta_{t}=5$ for the first 10 annotations, $\beta_{t}=-5$ for the next 10 annotations, and $\beta_{t}=0$ for the last 10 annotations. While five seems quite large for a regression coefficient, it is not large in relation to $\tau^{2}$, particularly the sparse settings which is what this article is concerned with. The variability of ψ is predicated on the amount of sparsity in the data and so large values for $\tau^{2}$ and $\beta_{t}$ are plausible in sparse situations.

Once $s_{j}$ is simulated, the $\sqrt{s_{j}}$ are used as coefficients for the true f. In this way, the $s_{j}$ imitate variable importance. A nonlinear true f is considered as $f\left(x_{i}\right)=0.5\sum_{j=1}^{p}\sqrt{s_{j}}{\tilde{f}}_{j}\left(x_{i,j}\right)+4.5$, where the final scaling and shifting is applied so that the simulated outcome approximates the observed gene expression outcome from ADNI. Because BART is designed to estimate nonlinear regression relationships, the individual ${\tilde{f}}_{j}\left(x_{i,j}\right)$ are nonlinear additive “pieces” to the overall function, defined below. There are 10 additive pieces that are recycled over each of the p SNPs so that ${\tilde{f}}_{1}\left(x_{i,1}\right)={\tilde{f}}_{11}\left(x_{i,11}\right)={\tilde{f}}_{21}\left(x_{i,21}\right)=\text{⋯}$ and ${\tilde{f}}_{2}\left(x_{i,2}\right)={\tilde{f}}_{12}\left(x_{i,12}\right)=\text{⋯}$, etc. In this way, function $f_{1}$ is the same as $f_{11},f_{21},\text{…}$ , and function $f_{2}$ is the same as $f_{12},f_{22}$, and so on. These 10 recycled functions, all of which exhibit nonlinearity, are

$$\begin{array}{cccccc} {\tilde{f}}_{1}\left(x_{i,1}\right) & = & \sin\left(\pi \left(x_{i,1}-1\right)\right) & {\tilde{f}}_{6}\left(x_{i,6}\right) & = & x_{i,6}^{2}-\sqrt{x_{i,6}}\\ {\tilde{f}}_{2}\left(x_{i,2}\right) & = & -x_{i,2}^{2} & {\tilde{f}}_{7}\left(x_{i,7}\right) & = & -\log\left(x_{i,7}+1\right)\\ {\tilde{f}}_{3}\left(x_{i,3}\right) & = & \text{exp}\left(x_{i,3}\right) & {\tilde{f}}_{8}\left(x_{i,8}\right) & = & \cos\left(\pi \left(x_{i,8}-1\right)\right)\\ {\tilde{f}}_{4}\left(x_{i,4}\right) & = & I\left(x_{i,4}<=1\right) & {\tilde{f}}_{9}\left(x_{i,9}\right) & = & -x_{i,9}\left(x_{i,9}^{2}-1\right)\\ {\tilde{f}}_{5}\left(x_{i,5}\right) & = & I\left(x_{i,5}>=1\right) & {\tilde{f}}_{10}\left(x_{i,10}\right) & = & I\left(x_{i,10}\neq 0\right)\sin\left(\pi \left(x_{i,10}-1\right)\right)\\ & & & & & -I\left(x_{i,10}=0\right)\sin\left(\pi \left(x_{i,10}-1\right)\right). \end{array}$$

A sample size of n=500 is used, approximated from the training data used in ADNI. An extra 1000 out of sample observations are simulated to evaluate the performance. Ten thousand MCMC samples were generated for each of the BART‐based methods with the first half being discarded for burn‐in and every fifth sample from the second half kept as a posterior sample for a total of 1000 posterior draws. Otherwise, default settings were used for the other BART settings as well as the other methods RF and SVR. The simulation study was performed over 500 data sets. Out‐of‐sample $R^{2}$ is used as the criteria for evaluating the performance of each method. The results are displayed in Tables 1 and 2 for strongly correlated SNPs and weakly correlated SNPs, respectively.

**Table 1:** Out of sample $R^{2}$ averaged over the 500 simulation results for all simulation settings with moderately or strongly correlated SNPs

Inform.	p	$\tau^{2}$	LN‐A	LN‐0	DART	BART	RF	SVR
No	100	1	35.21	35.85	35.65	36.63	31.48	29.42
No	100	5	41.85	42.14	41.84	41.96	39.40	33.39
No	100	10	42.03	42.51	42.29	40.25	39.98	30.43
No	250	1	35.14	35.60	35.24	37.32	34.82	33.19
No	250	5	37.57	38.12	37.45	39.81	37.74	36.63
No	250	10	40.17	40.33	40.15	37.70	31.70	23.55
No	529	1	31.35	32.32	31.11	33.30	32.69	31.28
No	529	5	37.28	38.39	37.38	38.62	34.52	32.40
No	529	10	47.80	47.69	47.45	44.27	46.48	17.09
Yes	100	1	46.23	46.02	46.02	42.02	42.63	31.62
Yes	100	5	51.01	50.74	50.66	45.88	46.84	28.71
Yes	100	10	48.63	48.40	48.40	44.27	45.12	32.62
Yes	250	1	48.16	47.62	47.48	42.88	44.63	35.60
Yes	250	5	50.74	50.39	50.23	46.85	44.25	42.47
Yes	250	10	48.29	47.76	47.61	42.56	44.96	35.31
Yes	529	1	47.39	46.41	46.00	42.59	44.09	35.70
Yes	529	5	47.46	45.99	45.90	45.20	42.69	43.82
Yes	529	10	45.91	45.05	44.68	41.34	43.15	33.94

*Note*: The first three columns give the simulation settings while the next six columns give the different methods. Informative means that the SNPs had informative annotations, that is, the coefficients in vector β≠0.

**Table 2:** Out of sample $R^{2}$ averaged over the 500 simulation results for all simulation settings for weakly correlated SNPs

Inform.	p	$\tau^{2}$	LN‐A	LN‐0	DART	BART	RF	SVR
No	100	1	32.76	33.47	33.00	35.33	23.51	23.50
No	100	5	39.30	39.51	39.46	36.59	33.53	23.78
No	100	10	40.87	40.92	40.98	37.46	34.74	21.36
No	250	1	21.77	22.76	22.03	25.74	14.64	15.51
No	250	5	32.02	32.29	31.73	29.29	25.91	13.91
No	250	10	44.06	43.99	44.14	38.16	32.63	15.56
No	529	1	9.79	11.61	9.59	13.46	7.13	7.82
No	529	5	21.61	21.93	21.50	20.03	15.77	8.10
No	529	10	38.11	38.15	37.74	30.49	35.79	8.91
Yes	100	1	48.44	48.05	48.07	42.50	46.73	25.27
Yes	100	5	47.73	47.19	47.24	41.87	46.69	25.24
Yes	100	10	50.21	49.81	49.81	44.02	47.20	25.46
Yes	250	1	49.62	48.89	48.73	42.49	47.20	15.73
Yes	250	5	46.77	45.89	45.78	39.31	45.58	15.21
Yes	250	10	47.34	46.48	46.43	40.45	47.41	15.43
Yes	529	1	48.23	46.48	46.40	39.18	44.38	12.26
Yes	529	5	50.34	48.85	48.60	42.59	48.23	12.09
Yes	529	10	48.70	47.23	47.19	41.14	46.32	12.18

*Note*: he first three columns give the simulation settings while the next six columns give the different methods. Informative means that the SNPs had informative annotations, that is, the coefficients in vector β≠0.

First, it appears that the BART‐based methods outperform RF and SVM across the simulation settings. Additionally, the logit normal prior without annotations is comparable or slightly better than DART, possibly as a result of the ability of the logit normal before take the correlation among the SNPs into account. This appears to be especially true when the annotations are uninformative. With uninformative annotations, the sparsity‐based methods appear to outperform standard BART in the extremely sparse setting where $\tau^{2}=10$, but do not outperform BART in the antisparse setting where $\tau^{2}=1$. The results from the moderately sparse setting, $\tau^{2}=5$, differ depending on whether the predictors are correlated or not. For $\tau^{2}=5$, the sparse methods do better when the simulated SNPs are weakly correlated. In the strongly correlated case, there is no clear winner. With informative annotations, the sparsity‐based methods appear superior in all cases. Finally, accounting for the annotations appears to offer a performance improvement when the annotations are informative while not hindering performance when the annotations are uninformative. This is particularly evident in the p=529 case, though the same pattern exists for p=250 and p=100.

## 5 GENOME‐WIDE SCAN USING ADNI

Data used in the preparation of this article were obtained from the Alzheimer's Disease Neuroimaging Initiative (ADNI) database (adni.loni.usc.edu). The ADNI was launched in 2003 as a public–private partnership, led by Principal Investigator Michael W. Weiner, MD. The primary goal of ADNI has been to test whether serial magnetic resonance imaging (MRI), positron emission tomography (PET), other biological markers, and clinical and neuropsychological assessment can be combined to measure the progression of mild cognitive impairment (MCI) and early Alzheimer's disease (AD). ADNI “is a longitudinal multicenter study designed to develop clinical, imaging, genetic, and biochemical biomarkers for the early detection and tracking of AD” (Mueller et al., 2005). Additionally, Shen et al. (2014) provide a review paper on ADNI for those interested.

Genome‐wide prediction (GWP) (Meuwissen et al., 2001) is becoming increasingly popular with many studies exploring the use of different prediction methods in a GWP setting (de los Campos et al., 2013; Howard et al., 2014; Okser et al., 2014), including BART (Waldmann, 2016). Many of these models emphasize sparsity which is a valuable property for a model in this situation because it is common for there to be a large number of SNPs relative to the sample size.

In this section, SNPs from the *cis*‐region of each gene are used to predict the genetic expression levels using standard BART as well as the sparsity‐based priors discussed in this article. This is an ideal setting to demonstrate the utility of these methods because certain genes may have a large number of SNPs in the *cis*‐region. Functional annotations are used to guide the selection of SNPs in the regression tree ensembles.

### 5.1 Prediction of gene expression using SNPs

Representing the genes are 45,040 probesets, all of them matching with one of 18,014 gene IDs. For each gene ID within chromosomes 1 through 22, the ADNI genotyping data was extracted from the *cis*‐region with 50,000 bp on either side of the region using the PLINK v. 1.07 software (Purcell et al., 2007). Any SNPs with an HWE *p* value less than 0.001 and minor allele frequency less than 0.01 (rare variants) were removed. Additionally, only SNPs with a genotyping rate greater than 0.1 were kept. Certain genes had no selected *cis*‐SNPs because there were no annotations measured for those variants. In total, 16,911 unique gene IDs were analyzed from the probesets.

Seven functional annotations (Pickrell, 2014) were extracted from the total set of annotations. These annotations include transcription start site distance (TSSdist) which is continuous along with binary indicators for nonsynonomous mutations, UTR3 and UTR5 exons, coding and noncoding exons, and K562 repressors. The SNPs were matched to the functional annotations and any SNP that did not have functional measured annotations were not used. This consisted of roughly 353,491, or 19.7%, removed from of the total 1,793,315 SNPs. If, for a given set of SNPs, any of these annotations had zero variation (i.e., only a single unique value), they were not considered for that gene.

Across these 16,786 genes, the mean and median number of cis‐SNPs (i.e., p) was 99.6 and 74, respectively while the first and third quartiles were 40 and 118. The average and median genetic length in kilo‐base pairs (kbp) was 60.0 and 21.9 kbp while the first and third quartiles was 7.0 and 60.2 kbp. For all data sets, the sample size is n=626.

A variety of methods were used for prediction: a null method, BART, DART, the logit normal prior without annotations (LN‐0), and the same logit normal prior with the set of seven annotations (LN‐A). For these methods, there were 1000 burn‐in MCMC iterations and another 800 MCMC iterations drawn across three chains. This yielded a total of 2400 draws from the posterior distribution. Though a smaller number of MCMC iterations are used in comparison to the simulation study, our model selection criteria (described in the next section) appeared to converge based on its Monte‐Carlo error. Therefore, this appeared to achieve reliable estimation while not being too computationally onerous.

The null method consists of fitting a Bayesian model with a null regression relationship for the SNPs. Only the random noise variance is estimated using an IG (0.01,0.01), or inverse‐gamma, prior. Comparing a predictive model with the results from this model evaluates the predictive ability of that model and is the strategy employed here. The annotation coefficients had an independent normal prior and each coefficient had a variance of 100 to make the prior uninformative. The variance of the annotation error, $\tau^{2}$, used a half‐T prior with scale 1 and degrees of freedom 3.

### 5.2 Bayesian model selection

While Pareto smoothed importance sampling (PSIS) can be applied and used with any importance sampling algorithm, Bayesian leave‐one‐out (LOO) cross‐validation is a major use case for it (Vehtari et al., 2017). Combining these two yields the acronym LOO‐PSIS. The output from LOO‐PSIS is the expected log predictive density, and is formally defined as $\text{ELPD}=\sum_{i=1}^{n}\log\left(p\left(y_{i}|y_{-i}\right)\right)$, where $y_{-i}$ represents the vector not including observation i. Computing the above for each i (and for each gene) would be computationally prohibitive, particularly with BART. However, importance sampling can be used instead. This is due to the following identity:

$$p\left(y_{i}|y_{-i}\right)={\left(E_{\theta |y}\left[1∕p\left(y_{i}|\theta \right)\right]\right)}^{-1}$$

where θ represents the parameters of a given model.

An intuitive estimator of the above quantity can be computed without refitting the model: ${\hat{\mu }}_{\text{MC}}=1∕\left(\frac{1}{W}\sum_{w=1}^{W}\frac{1}{p\left(y_{i}|\theta^{\left(w\right)}\right)}\right)$ where w=1,…,W indexes the MCMC iterations. However, the importance weights in this case are

$$p(y_{i}|\theta^{\left(w\right)})\propto p\left(y_{i}|y_{-i}\right)∕p\left(y_{i}|y\right)$$

and can be unstable which makes inference and asymptotics for ELPD difficult. This occurs when observation i is influential because the full posterior predictive distribution $p\left(y_{i}|y\right)$ is much different than the LOO posterior predictive distribution $p\left(y_{i}|y_{-i}\right)$ resulting in large or even infinite variance. In such cases, the required number of MCMC draws to achieve convergence with this estimator is much larger than W could practically be. Pareto smoothed importance sampling can be used to alleviate this issue, where the extreme values of the importance weights are smoothed with a three‐parameter generalized Pareto distribution (Vehtari et al., 2017). This distribution is commonly used to model tail behavior (Lee & Kim, 2019).

Additionally, the Pareto distribution gives a convergence diagnostic ${\hat{k}}_{i}$ for i=1,…,n, each of which speaks to the number of finite moments in the Pareto distribution. If ${\hat{k}}_{i}<0.5$, then both the first and second moment exist and so the usual Central Limit Theorem applies to the calculation of ELPD. Vehtari et al. (2017) suggest that ${\hat{k}}_{i}\in \left[0.5,0,7\right)$ can also be used if W, the number of MCMC samples, is large enough. However, the threshold is set to be the lower value of 0.5 in this analysis to ensure reliable ELPD estimates. When the value of ${\hat{k}}_{i}$ are too large for certain i, manual cross‐validation is performed on those observations to compute $\log\left\{p\left(y_{i}|y_{-i}\right)\right\}$ directly. For those i with ${\hat{k}}_{i}\leq 0.5$, the values of $\log\left\{p\left(y_{i}|y_{-i}\right)\right\}$ from LOO‐PSIS can be used. These estimates are then combined to yield a value for $\text{ELPD}=\sum_{i=1}^{n}\log\left\{p\left(y_{i}|y_{-i}\right)\right\}$ as desired.

Somewhat surprisingly, most samples in this analysis provide converging estimates of $p\left(y_{i}|y_{-i}\right)$ for all i and so the computational burden associated with cross‐validation is greatly reduced using LOO‐PSIS. Fundamentally, ELPD is an information criterion that depends on the likelihood of the model under examination and so its interpretation, outside of higher being preferable, is difficult. Therefore, the difference in ELPD is usually used to choose among competing models. In this case, these differences will be used to discover the genes whose *cis*‐SNPs are most predictive of their expression levels and also genes whose cis‐SNPs have informative functional annotations.

Note that we do not aim to develop a decision rule based on ELPD to select predictive genes and control for multiplicities in this small analysis which focuses on demonstrating the new logit‐normal prior. However, one well‐known way to control for multiplicities is based on controlling the false discovery rate, as in Muller et al. (2006). These ideas are not considered in this article, but incorporating them into larger analyses would be an interesting area of future work, especially for statistical genetics.

### 5.3 Genome‐wide results

The probeset with the largest ELPD difference between the null model and the standard BART model within a single gene ID is used as the probeset for that gene ID. Comparisons are made between the null model and standard BART to assess predictive ability. Additionally, comparisons are made between BART and the sparsity priors to assess their ability to aid in prediction, especially when the number of SNPs p is large. Finally, prior LN‐0 is compared against prior LN‐A, assessing the informativeness of the functional annotations. For genes with helpful annotations, the coefficients of these annotation models are presented.

**Figure 4:**
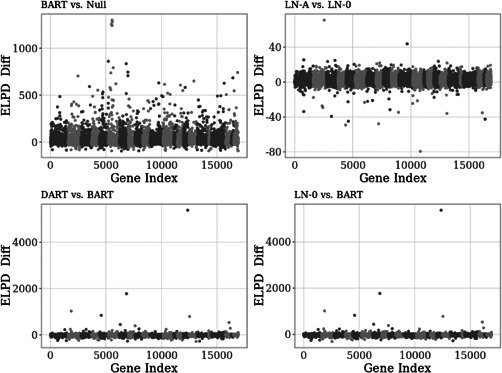
The ELPD difference for each model comparison is visualized in this figure with higher ELPD difference indicating preference to the more complicated model. The top‐left panel gives the results for the standard BART versus NULL comparison, the top‐right panel gives the results for the LN‐A versus LN‐0 prior, the bottom‐right panel gives the results for the LN‐0 prior versus BART, and finally the bottom‐left panel gives the results for DART versus BART.

First, standard BART is compared with the null model to ascertain genes whose expression levels have predictive SNPs in their *cis*‐region. The results for this comparison are shown in the top left corner of Figure 4. Each gene is indexed in terms of genomic position on the horizontal axis, while the vertical axis represents the ELPD difference between the null model and BART. From this, several genes appear to have predictive SNPs in terms of their expression levels. The top genes are summarized in Table 3, top‐left. The most predictive genes are those that belong to the human leukocyte antigen (HLA) system that is responsible for regulating the immune system. This system is known to affect many autoimmune diseases such as celiac disease (Martina et al., 2018), rheumatoid arthritis (Van Drongelen & Holoshitz, 2017), and type I diabetes (Noble, 2015). The relationship between the HLA system and autoimmune diseases is furthered in the literature by Aguiar et al. (2019). All of these genes were identified as possessing predictive *cis*‐SNPs at the genome‐wide significance level according to eQTLGen (Võsa et al., 2021), a large‐scale resource that incorporates 37 data sets to estimate the *p* values for the relationship between individual *cis*‐SNPs and the expression levels.

**Table 3:** Results from full genome scan presenting the five largest ELPD differences among the four model comparisons

BART versus null				LN‐A versus LN‐0			
Name	Chr	ELPD diff	*p*	Name	Chr	ELPD diff	*p*
HLA‐DPB1	6	1300.4	238	FN1	2	70.1	165
HLA‐DQB1	6	1280.0	171	ACTN3	11	43.8	49
HLA‐C	6	1256.3	365	F3	1	25.5	87
HLA‐DQA1	6	1243.0	114	CEP70	3	24.8	37
BTLN3	5	860.9	40	CRABP1	15	23.8	124
DART versus BART				LN‐0 versus BART			
Name	Chr	ELPD diff	*p*	Name	Chr	ELPD diff	*p*
CRABP1	15	5381.5	124	CRABP1	15	5370.0	124
LRGUK	7	1773.1	83	LRGUK	7	1769.4	83
CCDC85A	2	1024.5	350	CCDC85A	2	1014.0	350
RAB3C	5	842.3	302	RAB3C	5	827.3	302
MSLN	16	791.7	69	MSLN	16	781.7	69

*Note*: Although the ELPD difference for the DART versus BART and LN‐0 versus BART comparisons appear identical on the bottom, there are differences throughout the entire set of genes. However, these differences are small.

Next, the ability of the sparsity priors to aid in prediction is assessed by comparing both DART and LN‐0 to BART. The two bottom panels of both Figure 4 and Table 3 present these results. Certain genes appear to benefit from using sparsity priors, but the number of genes is reduced compared to the BART versus null comparison. Again, all of these genes contained predictive *cis*‐SNPs at a genome‐wide significance level according to eQTLGen. Gene FN1 has been implicated in endometreiosis (Pagliardini et al., 2013) and ACTN3 is well‐known to impact physical function (Pickering & Kiely, 2017).

There were a small number of genes with informative annotations found, as evidenced from the top right of Figure 4. The top genes from each model comparison are presented in Table 3. All of the genes listed have predictive *cis*‐SNPs according to eQTLGen. The annotation coefficients and their 95% credibility intervals are displayed in Table 4. Some of the annotations only have one unique value for all of the SNPs in the *cis*‐region of certain genes which is why some estimates are missing. Only the SNP with the highest ELPD contains a significant annotation at the 95% credibility level. However, this is unsurprising given the relatively small sample size of the ADNI data and does reflect the smaller ELPD difference for this comparison. When looking at the mean ELPD differences between the informative annotation prior and the sparsity priors (LN‐0 and DART), the mean difference across all 16,911 was 5.6 and 2.2, respectively. This indicates that the annotation prior may have been broadly useful across the genome, but the benefit was slight based on the mean ELPD differences. The *p* values of a paired *t*‐test in this case were miniscule (both being $<1\times 10^{-8}$).

**Table 4:** Posterior mean (95% credibility interval) for annotation coefficients

Name	Chr	ELPD diff	TSS dist	Nonsyn. mut.	UTR3 exons	UTR5 exon	Coding exon	Noncod. exon	K562 repressor
FN1	2	71.1		0.21 (−1.5, 1.8)	−1.95 (−17.7, 7.7)	−5.12 (−14.2, 0.7)	−4.27 (−14.0, 3.8)	−0.94 (−10.6, 7.9)	−1.81 (−21.4, 11.8)
ACTN3	11	43.8		0.60 (−2.5, 2.8)	**4.48** (**1.1**, **10.2**)	−**14.47** (−**26.8**, −**6.2**)	1.35 (−17.3, 10.3)		0.53 (−4.1, 7.3)
F3	1	25.5		−2.72 (−9, 3.1)		−1.05 (−3.5, 1.1)	3.79 (−1, 9.6)		
CEP70	3	24.8		−2.83 (−6, 1.2)	6.61 (−4.3, 15.6)	3.46 (−2.2, 8.9)	−7.81 (−24.1, 7.4)		−8.06 (−23.8, 6.9)
CRABP1	15	23.8	−0.69 (−13.1, 7.1)	−2.04 (−5.1, 0.1)	1.03 (−9.7, 7.7)	2.35 (−10.1, 13.8)			−0.80 (−16.5, 13.2)
RFTN1	3	23.6	−3.47 (−9.8, 5.6)	−0.33 (−1.6, 0.5)	9.72 (4.9, 15)	−1.62 (−6.9, 2.1)	0.33 (−6.8, 6)	−2.19 (−9, 5.6)	−8.28 (−13.9, −2.3)
LOC10…	8	22.0	4.68 (−8.8, 16.3)	−1.83 (−4.8, −0.2)	−0.12 (−7.3, 9.6)	−3.98 (−9.1, −0.7)	0.23 (−2.2, 5.1)	−4.48 (−19.4, 3.3)	1.18 (−16, 16.1)
CHST6	16	21.9		−0.07 (−6.5, 6.7)	−5.00 (−26.8, 11.8)	−1.29 (−9.6, 5.8)	−0.41 (−6.5, 5.8)	−2.53 (−17, 9.2)	−6.28 (−19.4, 7.4)
MICU3	8	21.0	0.68 (−21.0, 12.8)	−0.44 (−2.6, 0.7)	−5.70 (−24.6, 6.6)	−3.78 (−21.1, 6.4)	7.90 (5.5, 12.3)	−8.21 (−25.4, 0.9)	
KCNIP1	5	20.5	−5.05(−17.2, 2.9)	−0.56;(−4.0, 1.0)	6.24 (−8.0, 20.2)	−3.89 (−9.4, −0.7)		−1.05 (−10.3, 3.9)	6.14 (−7.2, 20.0)

*Note*: Some annotations have only one unique value for the cis‐SNPs of certain genes which is why some are missing in the below table. Only one of the genes has any significant annotations, in line with the relatively small ELPD differences for this comparison. Bold numbers imply that the CI does not cover 0, implying an informative annotation for that particular gene.

**Figure 5:**
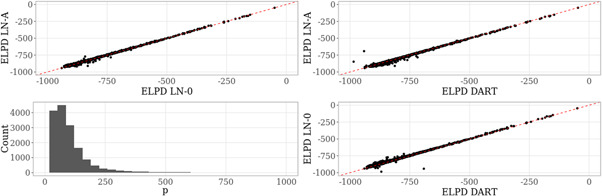
The ELPD values for each method are presented here. Dots higher than the red dashed line indicate genes that are more predictive using the model on the vertical axis. The top two panels indicate genes that may have informative annotations as given by ELPD. The bottom‐right panel shows the agreement between DART and LN‐0. Finally, the distribution of the number of *cis*‐SNPs *p* across the genome is presented in the bottom‐left.

Finally, it may be useful to compare the ELPD results from the different methods against each other. Doing this can ascertain any systematic differences between two of the methods. These results are presented in Figure 5. Points above the red line on the top row of this figure indicate genes that had informative annotations when compared to LN‐0 and DART on the left and right, respectively. This describes a small amount of the genes searched. Additionally, there are a small amount of genes lower than the red line, suggesting a possible loss of power when incorporating the annotations in select genes. Overall, however, there does appear to be significant agreement between LN‐0 and LN‐A for the vast majority of genes. Therefore, the authors recommend LN‐A unless it is known a priori that an annotation may not be informative, which is rare. The bottom right panel compares DART with LN‐0 and indicates that there are no systematic differences between these two priors as expected. The bottom left panel shows a histogram of the number of *cis*‐SNPs among the genes showing variety in the number of predictors considered.

## 6 DISCUSSION

Motivated by more informative and flexible modeling for gene expression prediction as shown in our real data example, this article has presented a novel logit normal prior for sparse modeling situations with Bayesian Additive Regression Trees that works as an alternative to the standard BART prior or the Dirichlet prior. This allows for prior information about the correlation structure to be incorporated, in contrast to the Dirichlet prior. Additionally, prior information about the predictors, such as the functional annotations in our real data example, can be seamlessly incorporated into the logit normal framework. Tractable MCMC inference can be performed using the PG augmentation strategy. Such annotations may not be widely informative as our analysis shows, but discovering informative annotations to filter the cis‐SNPs of a gene to predict its expression levels is a relevant question for statistical genetics.

In particular, as shown in our motivating real data example, prediction using SNPs is an area where such a prior is useful for several reasons. First, there are usually many SNPs compared to the number of observations and so a sparse prior can be beneficial. Second, the signals are weak in the sense that the effect sizes of the SNPs are typically small. Third, due to linkage disequilibrium, there are high correlations among the nearby SNPs. Such prior information could be incorporated to improve estimation in the presence of linkage disequilibrium. Hence, with the availability of many functional annotations on the SNPs, how to most effectively incorporate such informative priors into model building for prediction becomes both challenging and useful. Related to TWAS mentioned earlier, prediction can be particularly useful in the context of instrumental variable (IV) analysis, where SNPs can be used as instruments to draw causal inference about some pair of traits. Of course, this assumes that the IV assumptions are met and so the selection of SNPs as the instruments in the first stage becomes critically important for quality inference. Using BART to estimate a Bayesian nonparametric IV model with genetic data could be an interesting future avenue. Additionally, if the correlation structure of the predictors is known a priori, then it can be incorporated into the prior specification of ψ. This results in pairs of positively correlated predictors being selected together. Whether or not this results in robustness to the problem of predictor collinearity is an interesting topic to be explored in the future.

## Data Availability

The data that support the findings of this study are available from The Alzheimer's Disease Neuroimaging Initiative. Restrictions apply to the availability of these data, which were used under license for this study. Data are available from https://adni.loni.usc.edu with the permission of The Alzheimer's Disease Neuroimaging Initiative.
